# Effect of Weight Loss Medications on Hepatic Steatosis and Steatohepatitis: A Systematic Review

**DOI:** 10.3389/fendo.2020.00070

**Published:** 2020-02-21

**Authors:** Chelsea S. Pan, Takara L. Stanley

**Affiliations:** ^1^Metabolism Unit, Massachusetts General Hospital and Harvard Medical School, Boston, MA, United States; ^2^Pediatric Endocrine Division, Massachusetts General Hospital for Children and Harvard Medical School, Boston, MA, United States

**Keywords:** NAFLD (non-alcoholic fatty liver disease), steatohepatitis (NASH), obesity, weight loss medication, metformin, GLP-1 agonists, SGLT2 inhibitors, orlistat

## Abstract

Non-alcoholic fatty liver disease (NAFLD) is a common comorbidity in individuals with obesity. Although multiple pharmacotherapeutics are in development, currently there are limited strategies specifically targeting NAFLD. This systematic review summarizes the existing literature on hepatic effects of medications used for weight loss. Glucagon-like peptide 1 (GLP-1) agonists are the best-studied in this regard, and evidence consistently demonstrates reduction in liver fat content, sometimes accompanied by improvements in histological features of steatohepatitis and reductions in serum markers of hepatic injury such as alanine aminotransferase (ALT). It remains unclear whether these benefits are independent of the weight loss caused by these agents. Literature is limited regarding effects of orlistat, but a small number of reports suggest that orlistat reduces liver fat content and improves histologic features of NASH, benefits which may also be driven primarily by weight loss. A sizeable body of literature on hepatic effects of metformin yields mixed results, with a probability of modest benefit, but no consistent signal for strong benefit. There are insufficient data on hepatic effects of topiramate, phentermine, naltrexone, bupropion, and lorcaserin. Finally, a few studies to date suggest that sodium-glucose co-transporter-2 (SGLT2) inhibitors may reduce liver fat content and cause modest reductions in ALT, but further study is needed to better characterize these effects. Based on available data, GLP-1 agonists have the strongest evidence base demonstrating beneficial effects on NAFLD, but it is not clear if any weight loss medication has effects on NAFLD superior to those of nutritional modification and exercise alone.

## Introduction

Non-alcoholic fatty liver disease (NAFLD) is an increasing cause of morbidity, particularly in individuals living with obesity. Estimates of global prevalence are roughly 25% (1), with substantially higher prevalence in those with obesity (2). NAFLD includes simple steatosis as well as non-alcoholic steatohepatitis (NASH). Simple steatosis—accumulation of lipid within the hepatocyte—is generally thought to be benign with respect to liver health but contributes to metabolic dysregulation. NASH, characterized not only by steatosis but also by hepatic inflammation and hepatocellular damage, may develop into cirrhosis, hepatocellular carcinoma, and/or end stage liver disease. As in obesity itself, nutritional modification and increased physical activity are the foundation of treatment (2), but these are difficult to sustain. Although there is a robust pipeline of drugs in development for NAFLD, currently there are few established pharmacotherapeutic options specifically for individuals with NAFLD. Consequently, understanding the possible hepatic effects of currently used weight loss medications is important to aid clinicians in selecting appropriate therapy for individuals who desire weight loss medication and also have NAFLD. The current “gold standard” endpoints for improvement in NAFLD in clinical trials are either (A) resolution of steatohepatitis without worsening of fibrosis or (B) improvement in fibrosis without worsening of steatohepatitis, both requiring assessment by liver biopsy (3). None of the literature reviewed herein utilized these endpoints, which are specific to treatment trials of NAFLD. Rather, the trials reviewed either assessed liver histopathology from biopsy without the strictly defined endpoints above or, more commonly, reported surrogate measures of liver health such as changes in fat quantity or serum markers of liver inflammation (i.e., transaminases).

We present a summary of the current literature regarding hepatic effects of weight loss medications, achieved through a systematic review of the literature. The review includes drugs currently approved by the United States FDA for weight loss, as well as metformin and sodium-glucose co-transporter-2 (SGLT2) inhibitors (Table 1). Metformin is included because it is often used in individuals with obesity for modest benefits on weight loss or weight maintenance coupled with its other metabolic benefits, and SGLT2 inhibitors were included because this relatively new class of anti-diabetic medications is increasingly reported to have pleiotropic effects that include weight loss. This review only includes thiazoledinediones (TZDs, e.g., rosiglitazone or pioglitazone) when they are used as comparators for GLP-1 agonists, orlistat, metformin, or SGLT2 inhibitors. We excluded other thiazoledinedione studies because thiazoledinediones generally reduce liver fat content but cause modest weight gain rather than weight loss (2, 72). Dipeptidyl peptidase 4 (DPP-4) inhibitors also were not reviewed; these may result in modest weight reductions or be weight-neutral, but they are not generally used for the indication of weight loss.

**Table 1:** Characteristics of studies reviewed.

**References**	**Treatment^†^**	**Duration**	**Randomized?**	**Comparator(s)^†^**	**Blinded?**	**Sample size**	**Participant group**	**Biopsy performed?**	**Liver fat measure**
Anushiravani et al. (4)	Metformin 500 mg QD + LS	3 months	Y	1. LS + Placebo 2. LS + Silymarin 3. LS + Pioglitazone 4. LS + Vitamin E	Y	150	Adults, NAFLD	N	Not assessed
Armstrong et al. (5, 6) (LEAN)	Liraglutide 1.8 mg QD	48 weeks	Y	Placebo	Y	52	Adults, NASH, overweight	Y	Histology
Aso et al. (7)	Dapagliflozin 5 mg QD	24 weeks	Y	“Standard care” for T2D	N	57	Adults, T2D and NAFLD	N	Not assessed
Assy et al. (8)	Orlistat 120 mg TID + CR	6 months	N	(Open label)	N	14	Adults, obesity and NASH	Y	Histology
Aubuchon et al. (9)	Metformin 1,000 mg BID	6 months	Y	1. Clomiphene 2.Clomiphene+Met	Y	626	Adult women, PCOS	N	Not assessed
Bi et al. (10)	Exenatide 10 mcg BID	6 months	Y	1. Lispro 75/25 2.Pioglitazone	N	33	Drug-naïve adults with T2D	N	^1^H-MRS
Bouchi et al. (11)	Liraglutide 0.9 mg QD	36 weeks	Y	(Open label)	N	19	Adults, T2D on insulin, overweight	N	L:S ratio
Bugianesi et al. (12)	Metformin 2,000 mg QD	12 months	Y	1. Vit E 400 IU daily 2. LS	N	110	Adults, NAFLD, no T2D	Y*	Histology
Buse et al. (13) and Klonoff et al. (14)	Exenatide 10 mcg BID	2 years (13)/3 years (14)	N	(Open label extension phase report)	N	283	Adults, T2D	N	Not assessed
De Zegher et al. (15)	Metformin 425 mg BID × 2 years, then 850 mg BID × 2 years	4 years	Y	No treatment	N	34	Girls with LBW and precocious puberty	N	MRI
Duseja et al. (16)	Metformin 500 mg TID + LS	6 months	N	(Separate comparator group)	N	25	Adults, NAFLD and ↑ALT after 6 months LS + UDCA	N	Not assessed
Dutour et al. (17)	Exenatide 10 mcg BID	26 weeks	Y	“Standard care” for T2D	N	44	Adults, obesity + uncontrolled T2D	N	^1^H-MRS
Fan et al. (18)	Exenatide 10 mcg BID	12 weeks	Y	Metformin 500 mg BID (adjusted to max of 1,000 mg BID)	N	117	Adults, T2D and NAFLD	N	Not assessed
Feng et al. (19, 20)	Liraglutide 1.8 mg QD	24 weeks	Y	1. Metformin 1,000 mg BID 2. Gliclazide max 120 mg QD	N	87	Adults, T2D and NAFLD	N	U/S
Freemark et al. (21)	Metformin 500 mg BID	6 months	Y	Placebo	Y	32	Adolescents 12–19 years with obesity	N	Not assessed
Frøssing et al. (22)	Liraglutide 1.8 mg QD	26 weeks	Y	Placebo	Y	72	Adult women, PCOS	N	^1^H-MRS
Garinis et al. (23)	Metformin 500 mg BID	6 months	Y	LS alone	N	50	Adults, obese/ overweight and NAFLD	N	U/S
Gupta et al. (24)	Metformin 1,000 mg BID + LS	16 weeks	Y	Pioglitazone + LS	N	51	Adults, T2D	N	L:S ratio
Handzlik et al. (25)	Metformin 2,000 mg QD + LS	5 months	Y	LS alone	N	42	Adults, NAFLD	N	CAP
Harrison et al. (26)	Orlistat 120 mg TID + CR + Vitamin E	36 weeks	Y	Vitamin E + CR alone	N	50	Adults, NASH and overweight	Y	Histology
Harrison et al. (27)	Orlistat 120 mg TID	6 months	N	(Open label)	N	10	Adults, NASH and obesity	Y	Histology
Haukeland et al. (28)	Metformin 2,500 mg (3,000 mg if >90 kg) QD	6 months	Y	Placebo	Y	48	Adults, NAFLD	Y	Histology, L:S Ratio
Idilman et al. (29)	Metformin 850 mg BID	48 weeks	Y	1. LS + Rosiglitazone 8 mg daily 2. LS alone	N	74	Adults, NASH	Y	Histology
Ito et al. (30)	Ipragliflozin 50 mg QD	24 weeks	Y	Pioglitazone 15-30 mg QD	N	66	Adults, T2D and NAFLD	N	L:S Ratio
Kato et al. (31)	Metformin 500 mg QD	12 weeks	Y	Pioglitazone 15 mg QD	N	50	Adults, T2D	N	Not assessed
Kelley et al. (32)	Orlistat 120 mg TID + LS	6 months	Y	Placebo + LS	Y	39	Adults, T2D, overweight or obese	N	L:S Ratio
Kendall et al. (33)	Metformin 1,000 mg QAM+ 500 mg QHS	6 months	Y	Placebo	Y	151	Children 8–18 years with Obesity	N	Not assessed
Khoo et al. (34)	Liraglutide 3 mg QD	26 weeks	Y	LS	N	24	Adults, NAFLD and obesity	N	MRI
Krakoff et al. (35) (DPP)	Metformin 850 mg BID	Ave 3.2 year	Y	Placebo	Y	2,153	Adults at risk for T2D	N	Not assessed
Kuchay et al. (36)	Empagliflozin 10 mg QD	20 weeks	Y	“Standard care” for T2D	N	50	Adults, T2D and NAFLD	N	MRI-PDFF
Kusunoki et al. (37)	Various doses of 5 SLGT2 inhibitors and 5 DPP4 inhibitors	6 months	N	(Retrospective chart review)	N	214	Adults, T2D	N	Not assessed
Kusunoki et al. (38)	Luseogliflozin 2.5 mg QD	24 weeks	N	(Open label)	N	79	Adults, T2D	N	Not assessed
Lavine et al. (39) (TONIC)	Metformin 500 mg BID	96 weeks	Y	1. Vitamin E 400 IU BID 2. Placebo	Y	173	Children 8–17 years with NAFLD and ↑ALT	Y	Histology
Lingvay et al. (40)	Metformin 1,000 mg BID + insulin	3 months	N	(Open label)	N	19	Adults, newly diagnosed T2D	N	^1^H-MRS
Lingvay et al. (41)	Metformin 1,000 mg BID + Pioglitazone 45 mg QD + Glyburide <10 mg QD	Median 31 months	Y	Metformin 1,000 mg BID + aspart 70/30	N	16	Adults, treatment naive T2D	N	^1^H-MRS
Loomba et al. (42)	Metformin 2,000 mg QD	48 weeks	N	(Open label)	N	28	Adults, NAFLD, ↑ALT or AST	Y	Histology
Magalotti et al. (43)	Metformin 500 mg TID	24 weeks	N	(Open label)	N	20	Adults, NAFLD	N	Not assessed
Matikainen et al. (44)	Liraglutide 1.8 mg QD	16 weeks	Y	Placebo	Y	22	Adults, T2D and obesity	N	^1^H-MRS
Nadeau et al. (45)	Metformin 850 mg BID + LS	24 weeks	Y	Placebo + LS	Y	50	Adolescents 12–18 year, obesity and IR	N	U/S
Nair et al. (46)	Metformin 20 mg/kg QD	1 year	N	(Open label)	N	15	Adults, NAFLD, ↑ALT	Y*	Histology
Nar et al. (47)	Metformin 1,700 mg QD + LS	6 months	Y	LS alone	N	34	Adults, new T2D, NAFLD, and obesity	N	U/S
Nobili et al. (48)	Metformin 1,500 mg QD + LS	2 years	N	(Control group from separate study)	N	60	Children 9–18 years, NAFLD, overweight or obese	Y	Histology
Omer et al. (49)	Metformin 1,700 mg QD	12 months	Y	1. Rosiglitazone 4 mg daily 2. Rosi + Metformin	N	64	Adults, NAFLD, ↑ALT	Y	Histology
Petit et al. (50)	Liraglutide 1.2 mg QD	6 months	N	(Parallel insulin-intensification group)	N	80	Adults, uncontrolled T2D	N	^1^H-MRS
Preiss et al. (51)	Metformin 500 mg TID or 850 mg TID	8 months	Y	(Randomized to 1 of 2 metformin doses)	N	82	Adult women, PCOS and obesity	N	Not assessed
Resuli et al. (52)	Metformin 850 mg QD + LS	24 weeks	N	LS alone	N	61	Adults, NASH	N	Not assessed
Sabuncu et al. (53)^‡^	Orlistat + LS	6 months	?	Sibutramine + LS	N	25	Adults, NASH, obesity	N	U/S
Sanchez et al. (54)	Metformin 1,000 mg QD	12 weeks	Y	Exercise 60 min × 5 days per week	N	16	Adult women, overweight or obese	N	CT
Samson et al. (55) and Sathyanarayana et al. (56)	Exenatide 10 mg BID + Pioglitazone 45 mg QD	12 months	Y	Pioglitazone alone	N	21	Adults, T2D	N	^1^H-MRS
Schwimmer et al. (57)	Metformin 500 mg BID	24 weeks	N	(Open label)	N	10	Children <18y, NASH and obesity	N	^1^H-MRS
Shao et al. (58)	Exenatide 10 mcg BID + glargine	12 weeks	Y	Aspart + glargine	N	60	Adults, T2D, NAFLD, obesity, ↑ALT, AST, or GGT	N	U/S
Shibuya et al. (59)	Luseogliflozin 2.5 mg QD	6 months	Y	Metformin 1,500 mg QD	N	32	Adults, T2D and NAFLD	N	L:S ratio
Smits et al. (60)	Liraglutide 1.8 mg QD	12 weeks	Y	1. Sitagliptin 100 mg QD 2. Placebo	Y	52	Adults, T2D, overweight	N	^1^H-MRS
Sofer et al. (61–63)	Metformin 1,700 mg QD	4 months	Y	Placebo	Y	63	Adults, NAFLD	N	Not assessed
Sturm et al. (64)	Metformin 1,500 mg QD + pentoxifylline 4 mg TID + LS	48 weeks	Y	LS alone	N	19	Adults, NASH, ↑ALT	Y	Histology
Suzuki et al. (65)	Liraglutide 0.9 mg QD	24 weeks	N	(Open label)	N	59	Adults, T2D	N	CT
Tang et al. (66)	Liraglutide 1.8 mg QD	12 weeks	Y	Glargine	N	35	Adults, T2D inadequately controlled	N	MRI-PDFF
Tiikkainen et al. (67)	Metformin 1,000 mg BID	16 weeks	Y	Rosiglitazone 4 mg BID	Y	20	Adults, T2D drug naïve	N	^1^H-MRS
Torres et al. (68)	Metformin 500 mg BID + Rosiglitazone 4 mg QD	48 weeks	Y	1. Rosiglitazone alone 2. Rosiglitazone + 50 mg Losartan QD	N	137	Adults, NASH	Y	Histology
Uygun et al. (69)	Metformin 850 mg BID + LS	6 months	Y	LS alone	N	36	Adults, suspected NASH	Y	Histology
Yabiku et al. (70)	Metformin 1,000 mg QD	24 weeks	Y	1. Pioglitazone 30 mg QD 2. Sitagliptin 100 mg QD	N	886	Adult males, T2D and overweight	N	L:S ratio
Zelber-Sagi et al. (71)	Orlistat 120 mg TID	24 weeks	Y	Placebo	Y	52	Adults, NAFLD	Y*	U/S

1 H-MRS, Proton magnetic resonance spectroscopy; Bx, biopsy (Y indicates liver performed as part of the study; Y* indicates that it was performed for only a subset of participants); CAP, controlled attenuation parameter, a measure of liver fat by transient elastography/FibroScan; CR, caloric restriction; CT, computed tomography, assessing liver fat by some measure other than L:S ratio; IR, Insulin resistance; LBW, low birth weight; L:S ratio, liver-spleen ratio assessed by computed tomography; LS, Lifestyle modification, which includes any combination of the following: initial nutritional counseling, nutritional counseling throughout the study, prescribed caloric or macronutrient modification, prescribed exercise of any kind; MRI, Magnetic resonance imaging (with hepatic fat calculated by some method other than PDFF); MRI-PDFF, Magnetic resonance imaging—proton density fat fraction; Rand, randomized; T2D, type 2 diabetes; UDCA, ursodeoxycholic acid; U/S, ultrasound.

† Final/maximum doses are shown in cases where dosage was increased over the course of the study.

‡ *Only abstract was available, not full manuscript*.

## Methods

**Figure 1:**
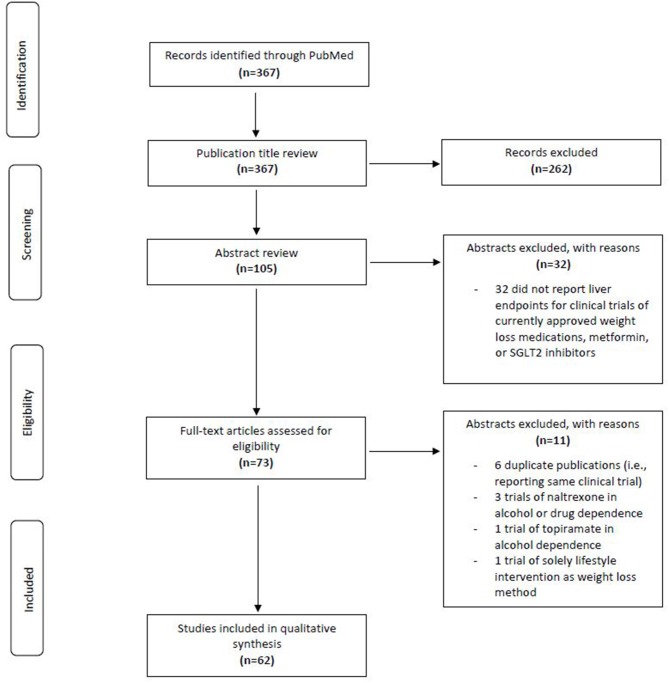
Preferred Reporting Items for Systematic Reviews and Meta-Analyses (PRISMA) diagram describing the selection of relevant publications for the review.

We aimed to review all publications in PUBMED reporting clinical trials of relevant medications that included endpoints of liver fat quantity, liver histopathology, and/or serum markers of liver inflammation, namely alanine aminotransferase (ALT), aspartate aminotransaminase (AST), or gamma-glutamyl transferase (GGT). Relevant medications included (1) medications that are currently FDA-approved for weight loss in the United States, (2) other glucagon like peptide 1 (GLP-1) agonists, given the known effect of the mechanism to reduce weight, (3) any sodium-glucose cotransporter-2 (SGLT2) inhibitor, given the known effect of medications in that class to reduce weight, and (4) metformin, which is often used for various indications in people with obesity. A PUBMED search without date restriction was performed on 9/6/2019 using the following search: (NAFLD OR non-alcoholic fatty liver disease OR AST OR ALT OR steatohepatitis OR non-alcoholic steatohepatitis OR NASH OR fatty liver) AND (metformin OR GLP-1 OR liraglutide OR exenatide OR semaglutide OR lixisenatide OR taspoglutide OR albuglutide OR bupropion OR topiramate OR orlistat OR lorcaserin OR phentermine OR naltrexone OR weight loss OR SGLT2) AND (Clinical Trial[ptyp]). There were no exclusion criteria or age limits included in the search. Figure 1 describes the process of publication selection. This search yielded 367 publications, of which review of the publication title yielded 105 potentially relevant publications. Upon further abstract review, 73 of these publications reported liver endpoints for clinical trials of currently approved weight loss medications, metformin, SGLT2 inhibitors, or lifestyle modification. After removing 6 publications that reported secondary results of a trial whose primary results were already represented in our abstract selection, as well as 4 publications describing naltrexone or topiramate for alcohol or substance dependence and one reporting results only of lifestyle modification, 62 publications remained. Table 1 shows the characteristics of the 62 studies investigating hepatic effects of GLP-1 analogs, metformin, orlistat, and SGLT2 inhibitors that were used in this review, and Table 2 summarizes the overall findings of the review.

**Table 2:** Summary of literature—effects on hepatic endpoints.

	**Histologic inflammation and fibrosis**	**Liver fat content**	**Serum markers of liver injury**
Bupropion	No data	No data	No data
GLP-1 agonists (20 publications available)	Insufficient data: 1 study with adequate duration measured using biopsy: higher resolution of NASH and lower progression of fibrosis compared to placebo (5)	Majority of studies show benefit over comparator, roughly 30–45% relative reduction	Majority of studies show modest reductions
Metformin (39 publications available)	Majority of studies show no benefit over comparator, but some suggest modest improvements in hepatic inflammation and hepatocellular injury. Very few suggest benefit to fibrosis	Majority of studies show no benefit over comparator, but some show modest reductions	Majority of studies show no benefit over comparator, with a few studies showing very modest reductions superior to lifestyle alone
Orlistat (6 publications available)	Insufficient data: 4 total studies, 3 showing no benefit over comparator (26, 27, 71), and 1 open-label study without comparator suggests benefit in both inflammation and fibrosis (8)	Insufficient data: 2 open-label studies without comparators suggest benefit (8, 27), whereas the 4 studies with comparators show no relative benefit (26, 32, 53, 71)	Insufficient data: 2 open-label studies without comparators suggest benefit (8, 27); 2 studies show no benefit over comparator (26, 53), and 1 study suggests benefit over placebo (71)
Naltrexone	No data	No data	No data for weight loss indication. From studies in alcoholism, seems to be neutral at low doses (≤50mg/d) but may be hepatotoxic at higher doses
SGLT2 inhibitors (6 publications available)	No data	Insufficient data: 2 studies with comparators [metformin and standard care] suggest benefit (36, 59) and 1 with pioglitazone comparator shows no relative benefit (30)	Insufficient data: 3 of 6 studies suggest modest benefit (7, 36, 38) and the other 3 suggest no benefit over comparator (30, 37, 59)
Topiramate	No data	No data	No data for weight loss indication. Benefit to lower ALT when used in alcoholism to reduce craving, otherwise no data

Studies that were double-blind with an active or placebo comparator were considered to be of highest quality. Given the variable natural history of NAFLD, a robust placebo response is present in studies of NAFLD and NASH (3, 73), such that studies without a comparator were considered to be of lowest quality. Optimal endpoints for studies of NAFLD and NASH are currently the subject of much discussion (3). This review considered (1) histological endpoints of steatosis, inflammation, hepatocellular damage, and fibrosis assessed via liver biopsy; (2) changes in liver fat content by various means including magnetic resonance proton spectroscopy (^1^H-MRS), proton density fat fraction (PDFF) by magnetic resonance imaging (MRI), liver:spleen ratio by computed tomography (CT), controlled attenuation parameter by transient elastography, or other ultrasound (U/S) or CT measures; and (3) changes in serum markers of liver injury, namely alanine aminotransferase (ALT), aspartate aminotransferase (AST), and gamma glutamyl-transferase (GGT). Currently MRS or MRI-PDFF are typically considered the best imaging modalities to quantify liver fat, whereas measures by CT or U/S are generally considered to have poorer performance than MRS or MRI. Controlled attenuation parameter (CAP) is a newer feature of transient elastography (i.e., FibroScan) that also assesses liver fat content.

Of note, studies investigating initiation of drugs in individuals with newly diagnosed and/or poorly controlled type 2 diabetes were reviewed but not used to determine magnitude of effects, as treatment with any anti-diabetic, including insulin, consistently improves liver fat compared to the untreated state.

## GLP-1 Agonists

GLP-1 agonists, initially approved by the FDA for the treatment of type 2 diabetes, repeatedly demonstrated significant effects to reduce body weight in clinical trials. Based on this effect, one drug in this class, liraglutide, has also been FDA approved for the indication of chronic weight management in patients with obesity or those with BMI ≥27 kg/m^2^ and weight related comorbidities (74). Of note, the FDA-approved dose for weight loss, 3 mg daily, is higher than the dose approved in T2D of 1.8 mg daily. GLP-1 agonists increase post-prandial secretion of insulin, decrease secretion of glucagon, slow gastric emptying, and increase satiety. Multiple publications have reported beneficial effects to reduce liver fat content and improve ALT, and one study has suggested a benefit in reducing progression of fibrosis (5, 10, 34, 50). Twenty publications reported the effects of a GLP-1 analog on liver-related endpoints; 12 of these investigated liraglutide (5, 6, 11, 19, 20, 22, 34, 44, 50, 60, 65, 66), and 8 investigated exenatide (10, 13, 14, 17, 18, 55, 56, 58). Four of these reported randomized, controlled, double-blind studies of liraglutide with a placebo (*n* = 3) or active (*n* = 1) comparator, and only one of these, the “Liraglutide Safety and Efficacy in Patients with Non-alcoholic Steatohepatitis (LEAN)” study, assessed histological endpoints using biopsy. Of note, of studies investigating liraglutide, only one (34) utilized a dose of 3 mg daily, whereas the remainder used 1.8 mg daily.

The LEAN study was a 48 week, multicenter, randomized, double-blind, placebo-controlled study assessing the efficacy of liraglutide 1.8 mg subcutaneously daily in 52 adults with NASH and BMI ≥25 kg/m^2^ (5). Seventeen patients had Type 2 diabetes. The study achieved the primary endpoint, demonstrating that 39% of patients in the liraglutide group compared to 9% in the placebo group met the primary endpoint of histological resolution of NASH without worsening of fibrosis (relative risk of resolution 4.3 [95% CI 1.0–17.7]) (5). Additionally, 36% of those on placebo had progression of fibrosis compared to only 9% of those receiving liraglutide (relative risk of worsening 0.2 [0.1–1.0]) (5). Steatosis improved in 83% of those receiving liraglutide vs. 45% of those on placebo (relative risk of improvement 1.8 [1.1–3.0]) (5). Gamma glutamyl transferase also decreased in the liraglutide group (−22.8 U/L [−40.4 to −5.2]), whereas ALT (−10.7 U/L [−25.9 to 4.5]) and AST (−6.7 U/L [−19.3, 5.9]) appeared to decrease more in the liraglutide group without achieving statistical significance. In the three other randomized, double-blind, placebo controlled trials of liraglutide, Matikainen et al. demonstrated 31% relative reduction in hepatic fat content by ^1^H-MRS in individuals with T2D who used a dose of 1.8 mg for 16 weeks (44). Frossing et al. demonstrated a 44% relative reduction in hepatic fat by ^1^H-MRS in women with polycystic ovary syndrome (PCOS) and BMI ≥ 25 kg/m^2^ who used a dose of 1.8 mg for 26 weeks (22). Smits et al. showed no change in hepatic fat content by ^1^H-MRS and no improvements in ALT, AST, or GGT in overweight patients with T2D taking liraglutide 1.8 mg daily for 12 weeks (60).

The majority of studies that investigated changes in hepatic fat content with liraglutide or exenatide demonstrated benefit, and all studies of 16 weeks duration or longer with steatosis measured via imaging as an endpoint demonstrated benefit. Magnitude of benefit was approximately 30-45% relative reduction in fat content in most studies (19, 22, 34, 44). Similarly, the majority of studies investigating serum markers of liver injury (ALT, AST, or GGT) demonstrated a benefit of liraglutide, with effect sizes ranging from quite modest (e.g., 5 U/L or less reduction in ALT) to ≥20 U/L and thus likely to be clinically meaningful. Importantly, baseline levels of these markers in a cohort determines possible magnitude of effect, such that cohorts with generally normal ALT, AST, and GGT at baseline are less likely to show improvement than those with substantial baseline elevations. Improvements in ALT and GGT ranged from 0 to 30 U/L in most studies, whereas improvements in AST tended to be more modest, usually ranging from 0 to 10 U/L (5, 10, 13, 14, 18, 19, 60).

An important unresolved question is whether GLP-1 agonists improve liver endpoints solely via effects on weight loss and improved insulin sensitivity, or whether there may be direct effects to improve hepatic steatosis or steatohepatitis. Only a few studies addressed this question. In the LEAN study, liraglutide achieved net effects of 4.4% body weight loss and 0.48% reduction in HbA1c, and changes in body weight and hemoglobin A1c were not different between those who had resolution of NASH and those who did not, potentially suggesting hepatic effects independent of weight loss or improvement in insulin sensitivity (5). Similarly, Buse et al. reported only modest associations between improvements in liver markers and improvements in weight or HbA1c (13). Physiologic mechanisms by which GLP-1 agonists may directly ameliorate NAFLD include both systemic and local anti-inflammatory actions (75), anti-oxidative effects (75), and amelioration of the endoplasmic reticulum stress response, resulting in decreased hepatocyte apoptosis (75, 76). Further research will be needed to elucidate how GLP-1 agonists may reduce steatosis and improve hepatocellular inflammation.

## Metformin

Although metformin's weight loss effects are modest, it also has glycemic benefits and is sometimes prescribed for both children and adults with obesity. Metformin has pleiotropic effects, and its multiple mechanisms of action are still not fully understood. It activates AMP-activated protein kinase (AMPK) in the liver, reducing hepatic gluconeogenesis, and also has multiple actions within the intestinal tract (77, 78). Thirty-nine publications meeting our search criteria reported on effects of metformin on liver endpoints (4, 9, 12, 15, 16, 18, 19, 21, 23–25, 28, 29, 31, 33, 35, 39–43, 45–49, 51, 52, 54, 57, 59, 61–64, 67–70), 10 of which were double-blind RCTs (4, 9, 21, 28, 33, 35, 39, 45, 61, 67). Only two of these investigated the effects of metformin on steatosis and steatohepatitis using liver biopsy before and after treatment (28, 39). In the TONIC trial, Lavine et al. randomized 173 children, ages 8–17 years, with NAFLD in a 1:1:1 ratio to metformin 1,000 mg daily vs. vitamin E 800 IU daily vs. placebo for 96 weeks (39). Metformin did not meet the primary outcome of greater percentage of patients with sustained reductions in ALT compared to baseline; this outcome was seen in 16% of metformin-treated patients and 17% of placebo treated patients (39). In secondary analyses, metformin did achieve greater reduction in hepatocellular ballooning score than placebo (−0.3 vs. +0.1), but it did not significantly affect any other histological endpoints (39). Forty-one percent of metformin-treated patients (95% CI 26–58%) had resolution of NASH during the study, compared to 28% of placebo-treated patients (95% CI 15–45%) (39). Similarly, Haukeland et al. showed no difference in the effects of metformin vs. placebo on histologic evidence of steatosis or steatohepatitis in 48 adults with NAFLD treated for 24 weeks, nor did they demonstrate differential effects on ALT reduction (28). As in the TONIC study, both placebo and treated groups improved, but improvements were not significantly different between groups (28).

Two additional double-blind studies investigated the effect of metformin on liver fat content as well as serum markers of hepatic inflammation (45, 67). Nadeau et al. randomized 50 obese adolescents ages 12–18 year in a 3:1 ratio to receive metformin 850 mg bid vs. placebo for 6 months, with all participants also receiving lifestyle education (45). In assessment of steatosis severity using liver ultrasound, metformin decreased steatosis relative to placebo (severity decreased by 0.5 points in the metformin group vs. a 0.35 point increase in the placebo group) (45). As in the previous studies, there were improvements in ALT, as well as GGT, in both metformin and placebo groups, with metformin not showing statistically significant benefit over placebo (45). Tiikkainen et al. compared metformin to rosiglitazone in 20 drug-naïve adults with T2D, using doses of 8 mg rosiglitazone daily or 2 g metformin daily for 16 weeks (67). In this study, Tiikkainen et al. did not find benefit of metformin to lower liver fat, whereas rosiglitazone achieved a relative reduction of 51% (67). Again there was no effect of metformin compared to placebo on serum ALT (67). Six double-blind RCTs reported effects of metformin on serum markers of liver inflammation (4, 9, 21, 33, 35, 61). Of these, five showed no difference in the sustained effects of metformin vs. placebo (*N* = 4) or clomiphene (*N* = 1) on these measures (4, 9, 21, 33, 61). In contrast, data from the Diabetes Prevention Program, which was a randomized controlled trial investigating the efficacy of lifestyle modification vs. metformin vs. standard care to prevent progression to T2D in adults with glucose intolerance, showed that the metformin group had a lower ALT compared to placebo over an average of 3.2 years of study participation (35).

In addition to the studies described above, multiple open-label studies compared metformin to a thiazolidinedione (24, 49, 70) or to lifestyle alone (12, 23, 25, 29, 47, 54, 64, 69) with regard to improvements in liver fat or inflammation measured by MRI/MRS, CT, US, fibroscan, or biopsy. The three studies directly comparing metformin to a TZD all showed benefit of the TZD to reduce liver fat (24, 49, 70), whereas only one showed a significant effect of metformin, albeit smaller in magnitude than the effect of TZD (70). Of 7 studies comparing effects of metformin vs. lifestyle on liver fat content (12, 23, 25, 29, 47, 54, 64), six showed positive effects of metformin to lower liver fat (12, 23, 25, 29, 47, 54), but, in all of these studies, metformin either was not clearly superior to lifestyle or was not directly compared to lifestyle. Similarly, in the 5 studies investigating changes in hepatocellular inflammation or fibrosis (12, 25, 29, 64, 69), metformin showed trends to modestly improve inflammation and/or fibrosis in 4 of them (12, 25, 64, 69), but none definitely demonstrated an effect that was superior to lifestyle alone. Overall, thirty-two studies included some data on changes in ALT, AST, and/or GGT (4, 9, 12, 16, 18, 19, 21, 23–25, 28, 29, 31, 33, 35, 39, 40, 42, 43, 45–49, 51, 52, 54, 57, 59, 61, 64, 67–69). Of these, 17 reported significant reductions in at least one of these measures in the metformin group (4, 12, 16, 19, 25, 28, 29, 35, 43, 45, 47, 48, 51, 52, 57, 68, 69), but metformin was superior to natural history or lifestyle comparators in only 5 of these (12, 16, 35, 52, 69), and in no cases was it superior to TZDs or GLP-1 agonists.

Overall, data on metformin in NAFLD suggest that it may have modest benefit compared to no treatment, but it is not consistently better than lifestyle modification alone. For those studies investigating histological changes, many report a very modest signal for improvement in inflammation and hepatocellular ballooning, but the overall evidence does not support a substantial effect of metformin on any measure of NAFLD.

## Orlistat

Orlistat is a gastric and pancreatic lipase inhibitor for obesity management that acts by preventing the absorption of dietary fats. Six studies have investigated the effects of orlistat combined with dietary counseling on liver-related endpoints (8, 26, 27, 32, 71). All reported improvement in liver fat content, while three showed improvement in histopathology by liver biopsy (8, 26, 71) and five showed reduction in inflammatory enzymes (8, 26, 27, 71). Two of these were randomized, controlled, and double-blinded studies of orlistat with a placebo comparator (26, 71), and only one of these, by Zelber-Sagi et al., assessed histological endpoints by biopsy.

Zelber-Sagi et al. conducted a 24-week, randomized, double-blind, placebo-controlled trial assessing the efficacy of 120 mg TID orlistat for the treatment of NAFLD in 52 adults (71). Participants were simultaneously enrolled in a behavioral weight loss program. Serum glucose and insulin levels were significantly higher in the orlistat group, which also presented with a higher degree of baseline fibrosis (71). All subjects received monthly evaluations of liver fat content by U/S, and 22 of the subjects (11 in each group) underwent biopsies at baseline and 24-weeks (71). The study demonstrated a significant decrease in serum ALT and AST levels, with an almost two-fold reduction in ALT in the orlistat group (48 vs. 26.4%) (71). Furthermore, there was significant reversal of fatty liver by U/S in the orlistat group only. Twenty-four percent of patients in the orlistat group (*p* = 0.04) had normal echogenicity after 24 weeks of treatment compared to 17.4% of patients in the placebo group (*p* = 0.08) (71). As measured through biopsy, the degree of steatosis and the degree of fibrosis improved in a comparable number of patients in both groups, and the effects did not reach statistical significance (71). Of note, these changes were seen in the context of similar weight loss in both groups, suggesting orlistat improved ALT and steatosis measured by U/S in NAFLD patients beyond its effect on weight (71). In the other randomized, double-blind, placebo-controlled trial of orlistat, Kelley et al. compared 120 mg TID of orlistat with placebo in patients with T2D also receiving behavioral weight loss intervention (32). They showed that liver fat content measured by liver-to-spleen ratio of CT attenuation values increased by 0.2 in a cohort whose baseline mean liver-to-spleen ratio was <1.0, the cutoff for representing fatty infiltration of the liver, in both the treatment and placebo groups (32). Unlike Zelber-Sagi et al., Kelley et al. suggest the decrease in fat content was a result of weight loss.

All studies using orlistat for a duration of at least 24 weeks showed improvement compared to baseline in hepatic fat content as well as levels of ALT and AST, but these changes were not consistently superior to other treatments such as lifestyle, sibutramine, or even placebo (8, 26, 27, 32, 71). Hepatic improvement was concurrent with 5–10% weight loss in all six studies (8, 26, 27, 32, 71) and several of the studies suggest that greater weight loss was associated with improved hepatic changes (8, 26, 27). Kelly et al. further suggest the additional benefit of orlistat as compared with generalized weight loss is the improvement in free fatty acid lowering and insulin sensitivity (32). Based on the relatively sparse current data, orlistat may have benefit for NAFLD insofar as it facilitates weight loss, but there is not clear evidence that it is superior to other methods or that it has effects on the liver independent of reductions in weight.

## SGLT2 Inhibitors

SGLT2 inhibitors, also known as gliflozins, inhibit the reabsorption of glucose in the kidney and thus lower blood sugar. They were developed for the treatment of type 2 diabetes mellitus (T2D), but recent literature suggests pleiotropic effects including potential cardiovascular benefit and modest weight loss (79, 80). Six studies have examined the effects of various SGLT2 inhibitors on patients with T2D and NAFLD (7, 30, 36–38, 59), but none to date have included histologic endpoints by biopsy. Five of the studies suggest improvement in at least one liver enzyme (7, 30, 36–38, 59), two showed improvement of hepatic fat content measured by liver-to-spleen ratio (30, 59), and one, the “Effect of Empagliflozin on Liver Fat in Patients with Type 2 Diabetes and Non-alcoholic Fatty Liver Disease (E-LIFT)” trial, showed improvement of hepatic fat content measured by MRI-PDFF (36).

The E-LIFT trial was a 20-week, randomized, controlled, unblinded study that compared standard treatment for T2D (i.e., anti-diabetic medicines other than SGLT2 inhibitors) plus 10 mg empagliflozin to standard treatment without empagliflozin among 50 adults with T2D and NAFLD (36). When included in the standard treatment for T2D, empagliflozin was significantly better at reducing liver fat measured by MRI-PDFF (36). The mean difference between empagliflozin vs. standard care was 4.0% greater reduction in absolute hepatic fat, or about 25% relative reduction, with empagliflozin (36). Four (18%) patients in the empagliflozin group achieved liver fat content <6% on MRI-PDFF compared with one (5%) in the control group (36). The two groups also showed significant differences for change in serum ALT with an effect size of 10.9 IU/L and reduced but non-significant differences for AST and GGT (36). Glucose and HbA1c decreased significantly in both groups; however, glycemic equipoise was maintained in the two groups by adjustment of other antidiabetic medications so that the changes in glycemic parameters had no effects on liver fat (36).

Two other 24-week studies comparing the SGLT2 inhibitors, luseogliflozin and ipragliflozin, with the active comparators, metformin and pioglitazone, showed moderate improvement in liver fat content measured by liver-to-spleen attenuation ratio (30, 59). The SGLT2 inhibitors also showed the effects of weight loss of about 1–2 kg and a decrease in HbA1c of about 1% (30, 59). Serum ALT, AST, and GGT improved in the ipragliflozin study but the change was not significantly different between the ipragliflozin and metformin groups (30). Histological measures were not made in either study. More data are needed on the effects of SGLT2 inhibitors on hepatic endpoints, particularly including biopsy, but early data suggest that they may reduce liver fat and achieve modest reductions in serum markers of liver injury. The mechanisms of these benefits is completely unclear at present, although modest weight loss as well as improvements in glycemia are potential mediators.

## Other weight-loss Medications

No publications were found in PUBMED investing the effects of topiramate, naltrexone, bupropion, phentermine, or lorcaserin on liver endpoints in individuals with NAFLD. In an abstract at the 2015 Meeting of the American Association for the Study of Liver Diseases (AASLD), Winokur et al. presented data on change in ALT in subjects who received naltrexone/bupropion (32 mg/360 mg) vs. placebo as part of three Phase 3 studies of naltrexone/bupropion, demonstrating among this large cohort that subjects in the lower 3 quartiles of ALT at baseline did not experience reductions in ALT on naltrexone/bupropion compared to placebo, whereas those in the 4th quartile of baseline ALT (i.e., highest 25% of values) had significant reductions in ALT when receiving naltrexone/bupropion compared to placebo (81). Similarly, in an abstract at the 2014 AASLD Meeting, Mehal et al. presented a retrospective analysis of three Phase 3 studies of lorcaserin, 10 mg twice daily, showing that those with high NASH clinical scores at baseline, indicating high risk for NASH, had greater improvements in ALT over 54 weeks of treatment (82). For both of these analyses, as for the studies previously discussed, it is unclear if there were effects of naltrexone/bupropion or lorcaserin independent of the weight loss achieved. Although no relevant publications were found for topiramate, studies investigating its use in individuals with alcoholism support safety with regard to topiramate and liver function (83).

## Conclusion

A summary of findings is presented in Table 2. The current body of literature suggests the GLP-1 agonists are likely the optimal choice for weight loss in someone with NAFLD/NASH, all other things being equal. Medications in this class have consistently demonstrated reductions in liver fat, and have frequently also shown benefits to reduce serum markers of liver injury. However, research is sorely lacking in other currently approved medications and medication-combinations that have weight loss effects similar to GLP-1 agonists (e.g., phentermine/topiramate and naltrexone/bupropion) and these studies need to be performed. Metformin has been extensively studied and, as is the case with many of its effects, evidence suggests the possibility of modest benefits but does not support any substantial improvements in NAFLD/NASH. Orlistat also shows a possible signal of benefit, but no studies with histological endpoints have been performed. SGLT2 inhibitors are an interesting emerging class with possibility of benefit, but, again, much additional research is required. Finally, most of the head-to-head comparisons of these medications with lifestyle modification alone do not demonstrate a clear superiority of pharmacologic therapy, such that it is unclear if any of these agents is better than effective lifestyle modification.

It is critical to note that most current studies are relatively small and are not blinded, and a fair percentage do not use randomization with active or placebo comparator arms. Further, the vast majority do not use gold-standard endpoints to determine effects on histologic features of NAFLD and NASH. Although reducing hepatic fat content is of great interest mechanistically, endpoints of inflammation, hepatocellular damage, and ultimately fibrosis are currently considered to be more clinically relevant, as these are the features that herald risk of cirrhosis and liver failure (3). Additionally, many of the studies were performed in patients with diabetes, sometimes in conjunction with addition of other anti-diabetic agents; in these studies, the effects of changing glycemia as well as any direct hepatic effects of other anti-diabetes medications will confound attempts to isolate the effects of the medication of interest. Another limitation of a review such as this is that, due to publication bias, review of available literature is likely to be biased toward “positive” findings. Additionally, “positive” studies with relatively small sample sizes routinely overestimate the true magnitude of effects (84). Thus, reviews such as this have unavoidable flaws, but hopefully serve to summarize existing literature and highlight needs in the field. Because of the substantial co-occurrence of obesity and NAFLD, understanding hepatic effects of weight loss medications is critical, and large, high-quality studies are needed to address this knowledge gap, particularly for agents for which few data are currently available.

## Author Contributions

TS conceived the project and performed the systematic literature review. TS and CP reviewed and summarized articles, wrote the manuscript, and edited and approved the final manuscript.

### Conflict of Interest

The authors declare that the research was conducted in the absence of any commercial or financial relationships that could be construed as a potential conflict of interest.
